# Multivariate decomposition of shift toward public facilities for inpatient care in rural India: evidence from National Sample Survey

**DOI:** 10.3389/fpubh.2025.1491297

**Published:** 2025-05-14

**Authors:** Sandeep Sharma, E Lokesh Kumar, Atul Kotwal

**Affiliations:** National Health Systems Resource Center, New Delhi, India

**Keywords:** utilization, public facilities, decomposition, inpatient care, rural India

## Abstract

**Introduction:**

Public facilities in health systems are essential for improving access and ensuring equity. Public facility utilization for inpatient care in rural areas increased between the most recent National Sample Survey (NSS) health rounds of 2014 and 2017–2018. This study conducted a decomposition analysis to identify the underlying causes that contributed to this increase in public facility utilization.

**Materials and methods:**

The study used the latest available unit-level data from the 2014 and 2017–2018 NSS Health Survey. The study employed multivariate decomposition analysis based on the existing behavioral model of access to health facilities.

**Results:**

The public facility utilization for inpatient care in rural areas increased from 41.6% to 45.3% between 2014 and 2017–2018. The results of the multivariate decomposition analysis indicate that differences in coefficients account for 81% of the increase in the utilization of public health facilities. Within the coefficients, this increase is mainly driven by the increase in the utilization of public facilities among those residing in states with relatively better public health systems (54.3%) and among the richest consumption class (45.4%).

**Discussion and conclusion:**

The utilization of public facilities for inpatient care increased between 2014 and 2017–2018 in rural India. This increase in utilization, though, was mostly driven by increased utilization among people residing in states with relatively better public health systems and by those belonging to the richer consumption classes. The study indicates that improved public health systems can play an important role in increasing footfall in public health facilities.

## 1 Introduction

The utilization of health services as a measure of access has been studied in detail (1, 2). However, the utilization of services from different types of providers, such as public (government-owned) or private, needs further elaboration in a country like India, which has a mix of both (3). Public facilities in India provide free or highly subsidized services, which is important both in terms of improving access and ensuring equity; the private providers, on the other hand, charge the patient for their services (4). The choice of public over private service or vice versa is largely a reflection of the quality of government facilities to meet the health needs of the population (5).

Rural India not only accounts for the majority of the population but also accounts for the majority of hospitalization cases (6). There has been a lot of emphasis by policymakers to improve public health services in rural areas. However, evidence from the last two decades on the utilization of inpatient care suggests that there has not been much improvement in terms of the utilization of public facilities. The share of public facilities in overall utilization for inpatient care was 44% in 1995–1996, which came down to 42% in 2004, and this share remained the same in 2014 (6). In 2017–2018, for the first time, there was a reversal in this trend, and the utilization of government facilities increased to 46% (6). Some of the prominent reasons for the decline in utilization of public facilities during the period 1995–1996 to 2004 include poor public health systems beset by problems such as inadequate allocation of resources, absenteeism, low-quality clinical care and low satisfaction among the public (7–9).

Given this change in trend over time, this paper aims to understand what factors explain this reversal in the trend of utilization of government facilities between 2014 and 2017–2018. Previous works on the choice of healthcare providers based on the National Sample Survey (NSS) data have mostly focussed on the trend and patterns of utilization and their implications on the cost of treatment (9–11). However, limited attempts have been made to unfold the underlying causes explaining changes in utilization over time and how different factors influence these changes (9–11).

The choice of a particular health service provider is determined by a complex interplay between patient and provider characteristics (12). The choice of health services between public and private providers can be understood in a broader context of Anderson’s model of access where the emphasis is on individual behavior and how the combination of predisposing, enabling, and need factors influence a person’s use of health services (13). A review of studies based on the model shows that age, education, gender, marital status, and employment status are predisposing factors whereas, income, medical insurance, and living location are the enabling factors (14). The need factors included chronic illnesses and perceived general health status (14).

Cross-country evidence on the utilization pattern between public and private health for 39 low-and middle-income countries using the World Health Survey data suggested that in the case of inpatient services, the poorest groups tend to rely almost exclusively on government facilities (15). Several studies have attempted to analyze the choice of provider between public and private providers in Indian contexts (16–19). One of the common findings of these studies was that individuals from the higher economic class were more likely to utilize private facilities than those belonging to the lower economic class, who relied more on public facilities (16–19). Certain other attributes such as affiliation to a certain social group, education, and age also played important roles in choosing a certain type of facility (16, 18, 19). Given this background, the objective of this study is to identify the reasons for the increase in the utilization of public health facilities in rural India between 2014 and 2017–2018.

## 2 Materials and methods

### 2.1 Data

The present study used the unit-level data of NSS Health and Morbidity rounds of the years 2014 (71st) and 2017–2018 (75th) (20, 21). The NSS is a large sample dataset and is nationally representative. The survey covers rural and urban areas spread across all the states and union territories (UTs) of the country. It collects information regarding morbidity, healthcare utilization, both inpatient and outpatient care, expenditure incurred for availing healthcare services from different care providers, the aging population, and information on households’ basic socio-economic characteristics.

Both surveys (NSS 71st and 75th rounds) use the same sampling design for the selection of households. In both rounds, it adopted a multi-stage stratified design for the survey. It had census villages and urban blocks as the first stage unit for rural and urban areas. In the ultimate stage, units were households in both sectors. The NSS covered 65,932 households and 333,104 individuals in 2014 and 113,823 households and 555,115 individuals in 2017–2018 (6, 22). In rural areas, it covered 36,480 households and 189,573 individuals in 2014 and 64,552 households and 325,883 individuals in 2017–2018 (6, 22). Although the sample size differed in these rounds, uniformity in sampling design in NSS allows for intertemporal comparison (23). Moreover, a uniform sample design permits the construction of comparable variables that could be used to make statistical inferences (24). Another important consideration while comparing estimates from the health survey data is to look at the effect of seasonality (25). The information about the hospitalization of a household member for inpatient care was collected for the reference period of the last 365 days before the date of the survey in both rounds. As a result, we have comparable data on hospitalization along with the type of hospital for both rounds. The analysis is based on data from rural areas of all the states and UTs and after dropping for missing values, the total number of observations used for analysis is 21,758 in 2014 and 35,519 in 2017–2018.

### 2.2 Dependent and independent variables

The dependent variable for our analysis is the utilization of inpatient care from different service providers (public or private) in the last year from the date of the survey. Here, we excluded hospitalization due to childbirth as this study aims to analyze the difference in health-seeking behavior based on an individual’s socioeconomic situation. Gender has been identified as one of the determinants when it comes to health-seeking behavior and the inclusion of hospitalization due to childbirth will hide the existing gender-based inequality (26). Demographic and socio-economic variables like age, social groups, education levels, gender, and economic condition are selected as independent variables for the study based on behavioral models of healthcare utilization established in different literature (16–19). Along with individual characteristics, enabling factors related to access are also selected as independent variables.

The age variable has four groups—less than or equal to 5, 6 to 14, 15 to 60, and above 60. Social groups are divided into two groups, viz. SC/ST and OBC/Others. The level of education has four categories-illiterate, up to primary, up to higher secondary, graduation & above while gender has two categories-male and female. To capture economic status, consumption class/quintiles based on monthly per capita consumption expenditure (MPCE) have been used. The MPCE was adjusted for household size and composition using the equivalence scale calculated as e_h_ = (A_h_ + 0.5K_h_)^0.75^ where A_h_ was the number of adults in the household and K_h_ was the number of children aged 0–14 years (27, 28). Consumption quintiles/class were then generated using the adjusted MPCE and were categorized as poorest, poor, middle, rich, and richest. To include the impact of the public health system in the states/UTs which might influence the choice for public facilities, individuals were divided into three categories: high-focus states, high focus-Northeastern states, and non-high-focus states (29). The high-focus states are the ones with weak public health indicators and/or weak health infrastructure (29, 30). The study defines the nature of ailments as a binary variable: communicable/other diseases. A similar classification was used in another study (24).

### 2.3 Statistical analysis

In this study, decomposition analysis has been used to understand the underlying factors explaining the shift toward public facilities for inpatient care in rural areas between the two time periods. Formal analysis has been performed using STATA version 14.1.

Decomposition analysis has been traditionally used to explain the wage differentials in the labor markets (31, 32). Lately, they have been employed for analysis of equity-related issues, particularly in the health sector (33–35). In India, various studies have employed decomposition analysis to understand the underlying causes of change in the critical issues related to the health sector of the country (36–39). Some of the common methods include the Oaxaca Binder decomposition technique and the Wagstaff decomposition technique (40–42).

In this study, Multivariate decomposition analysis based on logistic regression has been used to explain the increase in utilization of public health facilities in rural areas between 2014 and 2017–2018. The logistic regression coefficients were calculated using maximum likelihood estimation, and the regression is of the form:


$$\ln(\frac{P}{1-p})=\beta 0+\beta 1X1+\beta 2X2+\ldots +\mathrm{\beta n}\;\text{Xn}$$

Where *p:* probability of utilizing the public facility*, β_0_*: Constant, and *β_1_, β_2_,…, β_n_*: regression coefficients, *X_1_, X_2_, …, X_n_*: independent variables. The overall fit of the model was assessed using the likelihood ratio test.

The decomposition technique employed is based on the methods developed by Yun and is employed using the mvdcmp command developed for STATA by Powers et al. (43).

This technique allows us to decompose contributing factors for the change between two time periods.

The function form of the model is (43):


$$\begin{array}{l} {\bar{Y}}_{2017–18}-{\bar{Y}}_{2014}\;\\ =\underset{\text{Due}\;\text{to Difference in Characteristics}\;(E)}{\underset{︸}{\left\{\bar{F(X_{2017-18}\beta_{2017-18})}-\bar{F(X_{2014}\beta_{2017-18})}\right\}}}\;\\ +\{\underset{\text{Due}\;\text{to Difference in Coefficients}\;(C)}{\underset{︸}{\bar{F(X_{2014}\beta_{2017-18})}-\bar{F(X_{2014}\beta_{2014})}\}}} \end{array}$$

Where Y denotes the dependent variable, X denotes the independent variables, β denotes the coefficients, and F(∙) is the logistic function (43).

## 3 Results

### 3.1 Composition of public facility users

The sample weights of NSS were used to generate population-level estimates for the utilization of healthcare facilities. At the population level, around 42% of total hospitalization cases had taken place at public facilities in rural areas in 2014, while in 2017–2018 the share was around 45%. The rest of the cases were handled by the private sector. The utilization of public facilities is segmented based on individual characteristics as well as due to variations in the availability of services. A lesser percentage of those who are better off in terms of education, or economic conditions utilize public facilities as compared to those who are poor or less educated. Among the social groups, a higher share of vulnerable groups such as SC and STs utilize public facilities than their counterparts. In the case of disease conditions, those having communicable diseases rely more on public facilities. Individuals in high-focus states have a relatively higher dependency on public facilities as compared to their counterparts in non-high-focus states (Table 1).

**Table 1:** Composition in the utilization of public facilities for inpatient care among rural residents.

Variables	2014	2017–2018	Change
	Proportion (%)	Proportion (%)	
Overall	41.6 (41.6–41.7)	45.3 (45.3–45.4)	3.7
Social group			
OBC/others	36.4 (36.4–36.4)	40.7 (40.6–40.7)	4.3
SC/ST	54.2 (54.1–54.2)	56.8 (56.7–56.8)	2.6
Gender			
Male/Transgender	39.5 (39.5–39.5)	43.9 (43.8–43.9)	4.4
Female	43.8 (43.7–43.8)	46.9 (46.9–46.9)	3.1
Diseases			
Other diseases	40.2 (40.1–40.2)	42.9 (42.9–43)	2.7
Communicable diseases	45.4 (45.4–45.5)	50.1 (50.1–50.2)	4.7
Consumption quintiles			
Poorest	61.0 (60.9–61.0)	57.3 (57.3–57.4)	−3.7
Poor	54.2 (54.1–54.2)	50.3 (50.3–50.4)	−3.9
Middle	44.9 (44.9–45)	46.6 (46.5–46.6)	1.7
Rich	40.6 (40.6–40.7)	43.9 (43.9–43.9)	3.3
Richest	27.1 (27.1–27.1)	37.1 (37.0–37.1)	10.0
Age			
≤5	36.1 (36.0–36.2)	40.9 (40.9–41)	4.8
6–14	41.6 (41.5–41.6)	48.6 (48.5–48.6)	7.0
15–60	43.6 (43.6–43.7)	46.1 (46–46.1)	2.5
>60	37.5 (37.5–37.6)	42.9 (42.9–43.0)	5.4
Education level			
Illiterate	44.1 (44.0–44.1)	46.5 (46.5–46.6)	2.4
Upto primary	43.7 (43.7–43.8)	50.1 (50.0–50.1)	6.4
Till higher secondary/diploma	37.2 (37.2–37.3)	41.5 (41.5–41.6)	4.3
Graduation and above	27.7 (27.6–27.8)	27.2 (27.0–27.3)	−0.5
State categories			
High Focus-Northeastern States	89.3 (89.2–89.3)	85.6 (85.5–85.7)	−3.7
High Focus States	48.6 (48.5–48.6)	45.6 (45.6–45.6)	−3.0
Non-High Focus States	35.3 (35.3–35.3)	43.3 (43.2–43.3)	8.0

95% confidence interval are reported within parentheses; Change: Change between 2014 and 2017–2018; Authors’ calculations.

The change over time in the utilization pattern has not been uniform in all the groups. In the case of education level, there has been an increase barring the highest level where there is a slight decline. In the case of consumption class, the middle and higher groups have shown an increase, whereas for those in lower groups, there is a slight decline. Interestingly, those residing in non-high-focus states, or the better-off states have increased their use of public facilities, whereas those residing in high-focus states, there has been a decline in the usage of government facilities.

The odds of using public facilities based on logistic regression reiterate the point that the utilization of public facilities is segmented, as shown in Table 1. The odds of utilizing public facilities are much lower compared to the poorest with an adjusted odds ratio (AOR) of 0.4 (CI: 0.4, 0.5) for the richest and 0.5 (CI: 0.5, 0.6) for the rich consumption group in 2014 (Table 2). A similar pattern emerges even in 2017–2018. In the case of age, the likelihood of utilizing public facilities increases with age. Females are more likely to utilize public facilities both in 2014 with an AOR of 1.1 (CI: 1.0, 1.1) and in 2017–2018 with an AOR of 1.1 (CI: 1.0, 1.1). SCs/STs were more likely to avail services from the public facilities as compared to other social groups in 2014 with an AOR of 1.6 (CI: 1.5, 1.7) and in 2017–2018 with an AOR of 1.7 (CI: 1.6, 1.7).

**Table 2:** Odds of utilizing public facility for inpatient care in rural India-2014 and 2017–2018.

Independent variables	2014		2017–2018	
	OR	*p* value	OR	*p* value
SC/ST (Ref: Others/general)	1.605 (1.508–1.708)	0.000	1.652 (1.574–1.734)	0.000
Female (Ref: Male/Transgender)	1.069 (1.007–1.135)	0.028	1.057 (1.010–1.106)	0.018
Communicable diseases (Ref: Other diseases)	1.366 (1.279–1.459)	0.000	1.569 (1.494–1.648)	0.000
Age (Ref: ≤5)				
6–14	1.220 (1.049–1.418)	0.010	1.292 (1.150–1.452)	0.000
15–60	1.425 (1.274–1.594)	0.000	1.458 (1.330–1.599)	0.000
>60	1.417 (1.254–1.601)	0.000	1.459 (1.321–1.612)	0.000
Education level (Ref: Illiterate)				
Upto Primary	1.018 (0.941–1.102)	0.654	1.095 (1.029–1.165)	0.004
Upto Higher Secondary/Diploma	0.834 (0.771–0.902)	0.000	0.826 (0.778–0.877)	0.000
Graduation and above	0.511 (0.422–0.619)	0.000	0.492 (0.431–0.561)	0.000
Consumption quintiles (Ref: Poorest)				
Poor	0.822 (0.741–0.912)	0.000	0.754 (0.695–0.819)	0.000
Middle	0.699 (0.632–0.772)	0.000	0.718 (0.662–0.778)	0.000
Rich	0.543 (0.490–0.601)	0.000	0.682 (0.630–0.739)	0.000
Richest	0.410 (0.372–0.452)	0.000	0.566 (0.525–0.611)	0.000
State categories (Ref: High focus-northeastern states)				
Non-high focus states	0.113 (0.100–0.127)	0.000	0.143 (0.131–0.157)	0.000
High focus states	0.160 (0.142–0.181)	0.000	0.191 (0.175–0.210)	0.000
Constant	5.337 (4.475–6.363)	0.000	4.217 (3.671–4.844)	0.000
Likelihood ratio test				
LR chi2(15)	3188.14	0.000	4417.35	0.000

OR, Odds Ratio; 95% Confidence Interval are reported within parentheses. Authors’ calculations. The results of the likelihood ratio test indicated that the model is significantly better fit than the null model in both the years.

The utilization of public facilities is more likely in the case of patients suffering from communicable diseases for both years. Among those residing in non-high-focus states and high-focus states, the likelihood of utilizing public facilities was less than those residing in northeastern high-focus states in both years.

### 3.2 Decomposition analysis

Decomposition analysis of the difference in utilization of public facilities in the two time periods can be divided into two parts-due to differences in characteristics and due to differences in coefficients. The differences in characteristics indicate the change in utilization of public facilities due to compositional changes in the population between the two periods. The differences due to coefficients indicate the change in public facility utilization because of behavioral changes in the population between the two periods (43, 44).

The results show that differences in the coefficients/effects account for 81%, while differences in characteristics or endowments account for 19% of the observed difference in the utilization of public facilities in rural areas between 2014 and 2017–2018 (Table 3). This finding indicates that between the two periods, the change in utilization of public facilities is mostly due to a higher propensity of utilizing public facilities in 2017–2018 than in 2014.

**Table 3:** Multivariate decomposition of the change in utilization of public facilities for inpatient care in rural areas between 2014 and 2017–2018.

Variables	Due to difference in characteristics (E)			Due to difference in coefficients (C)		
	Coef.	*p* value	Contribution (%)	Coef.	*p* value	Contribution (%)
SC/ST (Ref: Others/general)	0.001 (0.001–0.001)	0.000	**1.3**	0.002 (−0.004–0.008)	0.475	**4.5**
Female (Ref: Male/Transgender)	−0.000 (−0.000–−0.000)	0.018	**−0.1**	−0.001 (−0.009–0.007)	0.766	**−2.6**
Communicable diseases (Ref: Other diseases)	0.004 (0.004–0.005)	0.000	**8.5**	0.009 (0.004–0.015)	0.001	**19.6**
Age (Ref: ≤5)			**2.1**			**11.6**
6–14	0.001 (0.001–0.001)	0.000	1.9	0.001 (−0.003–0.005)	0.551	2.3
15–60	0.000 (0.000–0.001)	0.000	1.0	0.003 (−0.018–0.024)	0.756	7.0
>60	−0.000 (−0.001–−0.000)	0.000	−0.8	0.001 (−0.005–0.007)	0.717	2.3
Education level (Ref: Illiterate)			**−4.9**			**7.8**
Upto Primary	0.000 (0.000–0.000)	0.005	0.3	0.004 (−0.002–0.011)	0.158	9.5
Upto Higher Secondary/Diploma	−0.002 (−0.002–−0.001)	0.000	−3.4	−0.001 (−0.007–0.006)	0.857	−1.2
Graduation and above	−0.001 (−0.001–−0.001)	0.000	−1.8	−0.000 (−0.002–0.001)	0.747	−0.5
Consumption quintiles (Ref: Poorest)			**−1.1**			**63.2**
Poor	0.000 (0.000–0.000)	0.000	0.2	−0.003 (−0.008–0.002)	0.202	−6.5

Middle	0.001 (0.001–0.001)	0.000	2.1	0.001 (−0.005–0.007)	0.681	2.6
Rich	−0.001 (−0.001–−0.001)	0.000	−1.6	0.010 (0.005–0.016)	0.001	21.7
Richest	-0.001 (−0.001–−0.001)	0.000	−1.8	0.022 (0.013–0.030)	0.000	45.4
State categories (Ref: High focus—northeastern states)			**13.3**			**87.5**
Non-high focus states	0.007 (0.007–0.008)	0.000	15.1	0.026 (0.008–0.044)	0.004	54.3
High focus states	−0.001 (−0.001–−0.001)	0.000	−1.8	0.016 (0.001–0.030)	0.031	33.2
Constant				−0.053 (−0.105–−0.001)	0.046	−110.6
Overall	**0.009** (0.008–0.010)	**0.000**	**19**	**0.039** (0.031–0.047)	**0.000**	**81**

95% confidence intervals are reported within parentheses. Authors’ calculations.

Bold values in the contribution columns indicate the sum of contribution of each categories.

#### 3.2.1 Difference in characteristics

Overall, the difference due to change in characteristics accounted for 19% of the difference in the utilization of public facilities in rural areas between 2014 and 2017–2018. The significant contributors to the difference in characteristics are the type of state a person is residing in-high focus/non-high focus (13.3%), and the type of disease-communicable diseases/other disease (8.5%).

#### 3.2.2 Difference in coefficients

The difference in coefficients accounted for the most difference observed in the utilization of public facilities in rural areas between 2014 and 2017–2018. The major contributors to this change are changes in the effects of the type of state a person is residing in high focus/non-high focus (87.5%), consumption quintiles (63%), and those reported suffering from communicable diseases (20%). Among the different areas of regions, the change in the effects of those residing in developed states or non-high-focus states is the major contributor (54%). Among the different consumption quintiles, the major contributor is the change among the richest quintile class (45%). Other factors like education levels, age, social group (SC/ST or others), and gender contribute a relatively smaller proportion to the difference in utilization of public facilities between the 2 years.

These findings highlight the important factors contributing to the increase in the utilization of public facilities in rural areas between 2014 and 2017–2018.

## 4 Discussion

The health system in India is characterized by a mixed system wherein both public as well private providers play an important role in health service delivery. Though health planning in India was rooted in the public provision of health services, private participation, especially for inpatient care, which is resource-intensive, has been part of the government’s policy document (45). One of the primary reasons for the utilization of private facilities is the perception of better quality even though the cost is much higher in private facilities as compared to public (5, 6, 22). In a country like India choice of private over public facilities not only indicates a failure of government facilities but also has serious financial implications for the households.

The available trend on utilization in rural areas of the country where the majority of the population revealed a gradual shift toward the private provider over time from 56% in 1995–1996 to 58% in 2014 (6). But there has been a reversal in trend between 2014 and 2017–2018 and there has been an increase in the use of public facilities for inpatient care from 42 to 46% (6). Although this change in utilization pattern has been reported, to the best of our knowledge, this is the first study to understand this change in a more holistic manner taking into account important determinants that influence the use of public facilities and how it has changed over time to influence this shift.

This paper investigated the factors causing the increase in the utilization of public facilities for inpatient care between 2014 and 2017–2018 using the health survey of NSS. The utilization pattern suggests that the poor, less educated, socially vulnerable groups (SCs and STs) are more likely to utilize public health facilities as compared to their counterparts. Utilization was higher among those residing in the northeastern states as compared to others.

Decomposition analysis was undertaken to understand and analyze how the different covariates influenced the change in the utilization pattern. The multivariate decomposition technique was used to understand how these factors contributed to this increase in the utilization of public facilities. The analysis indicates that this increase is mainly driven by the rise in utilization of public facilities in non-high-focus states and among the rich. Other studies have pointed out that there was an increase in the utilization of public facilities in non-high-focus states between 2014 and 2017–2018 (11). The increase in the utilization of public facilities in non-high-focus states indicates that states with relatively better public health systems are better placed to improve access to public facilities than the other states. The government health expenditure has increased between the two periods (11, 46). However, despite the increase, the findings of this study indicate the benefits are not evenly distributed across the states as the effectiveness of this expenditure depends greatly on the existing public health system (11, 46).

The decomposition analysis further reveals that the increase in the utilization of public facilities by the economically richer groups is another important contributory factor adding to this increase. Despite the utilization of public facilities among the poorer classes remaining above 50% in both rounds, the decline observed between 2014 and 2017–2018 presents a complex issue as the poor are more financially vulnerable (8). Though this decline in utilization among the poorer sections needs detailed analysis, one plausible reason for the decrease in utilization of public facilities among the poorer sections and the increase in utilization by the richer section could be the relatively higher concentration of the richer section of the country’s population in non-high focus states and higher concentration of poorer sections in high focus states.

One of the limitations of this study is related to people’s perception of services. The NSS Health Survey does not incorporate people’s perception of the quality of services that determine their choice. It does not provide information to empirically test how perception determines the choice between public and private health services. Although the survey does ask about the reasons for not going to government health facilities, this question is limited to only those going for private services (6, 22).

## 5 Conclusion

Inpatient care is an important part of curative care and is integral to any health system. Though health planning in India was rooted in the public provision of health services, there has been a rapid growth of private healthcare in the country over the years. Even in rural areas, there has been a decline in the utilization of public facilities in the last two decades. The recent shift toward public facilities between 2014 and 2017–2018 can be considered a reversal in this trend. This study provides the reason for this shift in the utilization of inpatient care from public facilities using the available behavioral model on access to health. The findings suggest the shift observed between 2014 and 2017–2018 is mostly due to increased utilization of public facilities among those residing in non-high-focus states and by the richer section of the population. These findings signify that good public health systems are a basic necessity for improving access to healthcare services.

## Data Availability

The original contributions presented in the study are publicly available. The unit level data can be found here: https://microdata.gov.in/NADA/index.php/home.
